# Decarbonizing the cementitious materials cycle: A whole‐systems review of measures to decarbonize the cement supply chain in the UK and European contexts

**DOI:** 10.1111/jiec.13105

**Published:** 2021-02-03

**Authors:** Sarah Pamenter, Rupert J. Myers

**Affiliations:** 1 School of Engineering, University of Edinburgh, Edinburgh, UK; 2 Department of Civil and Environmental Engineering, Imperial College London, Skempton Building, London, SW7 2AZ UK

**Keywords:** cement, circular economy, decarbonization, emission reduction, industrial ecology, policy analysis

## Abstract

This paper presents a semi‐quantitative review of measures to achieve net‐zero greenhouse gas emissions (“decarbonization”) in the cementitious materials (CMs) cycle, that is, activities directly related to cement spanning extraction through to end of life. It focuses on the United Kingdom and Europe in order to relate these measures, comprising emissions, energy, and material efficiency, to the policy landscape. We summarize our findings in an annotated CMs cycle, produced by reconciling the diverse yet relatively underdeveloped literature on the topic, to quantify decarbonization potentials of the various measures in a systematic manner. We find that decarbonization measures with significant potential exist along the entire CMs cycle, although upstream (of use), energy, and emission efficiency measures are better quantified than downstream (of use) and material efficiency measures. Notably, the decarbonization potentials of recycling technologies and the ways in which technological advancements may transform the CMs cycle and thus the stocks, flows, and processing of materials, as well as effectiveness of decarbonization measures, are poorly understood. Therefore, this paper provides a basis to systematically understand the effects of emissions, energy, and material efficiency measures on decarbonization of the CMs cycle and, in this context, the interplay between technology, economic actors, and policy. This article met the requirements for a gold–gold JIE data openness badge described at http://jie.click/badges.

**Supplementary Information:**

The online version of this article (doi:10.1111/jiec.13105) contains supplementary material, which is available to authorized users.

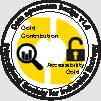

## INTRODUCTION

Industrial greenhouse gas (GHG) emissions (hereafter “emissions” or “industrial emissions” where further clarity is needed) accounted for ∼30% of the global total in 2010 (Bajželj, Allwood, & Cullen, 2013; IPCC, 2014). To limit global warming to 1.5°C above pre‐industrial levels, as per the Paris agreement (UNFCCC, 2015), industrial emissions are required to reduce by 54−91% (relative to 2010 levels) by 2050 (IPCC, 2018). The cement industry accounted for ∼23% of industrial emissions globally, or ∼8% of total global emissions, in 2012 (Favier, Scrivener, & Habert, 2019; IEA, 2009; IEA, 2018; Miller, Horvath, & Monteiro, 2016; WWF, 2008). As with total industrial emissions, emissions from the cement industry are projected to increase under a business‐as‐usual scenario, due to rising global demand for cementitious material (CM) products (i.e., cement, mortar, concrete) (IEA, 2018; WWF, 2008). Therefore, decarbonizing the cement industry, here defined as reducing its emissions to net zero, presents a significant challenge. This challenge is technical, for example, due to the high energy and emissions intensities of the production processes, and economic, for example, due to the globalized and relatively low‐profit‐margin nature of CM products (Bataille et al., 2018). Careful policy intervention is thus essential in decarbonizing the global cement industry, and other industries which share similar challenges. This is particularly relevant in high‐income countries, where development and deployment of low‐emissions technologies must be stimulated while avoiding “carbon leakage” (Forin, Radebach, Steckel, & Ward, 2018; Grubb, Hope, & Fouquet, 2002).

Higher‐income countries, like the UK, have historically focused emissions‐related policy on promoting energy efficiency (e.g., cement kiln efficiency) and “emissions efficiency” (e.g., carbon capture and storage/utilization, CCS/U) at the cement production stage (BEIS, 2017a; DECC, 2015a; EEA, 2019). This focus matches that of research into decarbonizing the cement industry, which has historically focused on energy and emissions efficiency measures implemented in the Portland cement (PC) clinker production process (BEIS, 2017a; CEMBUREAU, 2013; IEA, 2018; Lehne & Preston, 2018; WWF, 2008). However, the cost of further emission reductions through such measures can be high, for example, CCS (which would result in a 70–115% increase in cost per tonne cement produced; Material Economics, 2019), and rates of implementation can be slow. Expanding the focus of emissions‐related policy from the production stage to the whole CMs cycle can mitigate this problem. Recent studies highlight the following benefits of such a systems approach: reduced cost, less urgency for technological development, and a wider, less concentrated distribution of effort (Favier, De Wolf, Scrivener, & Habert, 2018). Notably, a systems approach includes implementation of material efficiency measures.

Material efficiency has been studied for at least a decade (Allwood, Ashby, Gutowski, & Worrell, 2011), and is here defined to include product‐service efficiency and demand reduction. Recent studies have quantitatively demonstrated the significant potential of material efficiency to reduce emissions in industries like cement (European Commission, 2018; Favier et al., 2018; Hertwich et al., 2019; Material Economics, 2019; Shanks et al., 2019). While the potential of material efficiency is also acknowledged in UK government literature, notably in recent policy strategy under a broader “circular economy” context (DEFRA, 2018, 2020), it has historically received little attention in published policy or industrial strategy (DECC 2015a, 2015b; BEIS 2017b), which risks missing opportunities to decarbonize industry in the most efficient way. This omission could partly be explained by mismatch in the scopes of policy and material efficiency literature. Generally, policy is industry and country specific, whereas material efficiency studies leverage systematic material cycle and/or life cycle perspectives. Material efficiency also receives less research literature coverage relative to energy and emissions efficiency.

In this paper, we review the state of knowledge on the potentials of emissions, energy, and material efficiency measures to decarbonize the CMs cycle. We collate the relatively small and disparate literature to facilitate a consistent and comprehensive overview (“snapshot”) of the topic. Thus, we address the main limitation of studies to date in this literature, their lack of a comprehensive, succinct, and/or accessible synthesis of decarbonization measures along the entire CMs cycle. For example, Shanks et al. (2019) focus on material efficiency measures and exclude specific analysis of the end‐of‐life stage in the CMs cycle, while Material Economics (2019) and Favier et al. (2019) present reports that are long and not directly comparable. We present our overview in a concise and annotated visualization of the CMs cycle, including processes, emissions, potential decarbonization measures, and relevant actors along it. This visual summary facilitates an understanding of what the most important decarbonization measures are, where the key intervention points for policy are, and where the gaps between the research literature and practical implementation lie. Further analysis of this annotated CMs cycle highlights the relative significance of material efficiency measures and whether they are targeted in current UK/European industrial decarbonization policy, or otherwise.

Overall, we aim to clarify the significance of material efficiency measures in the CMs cycle and better understand how industrial decarbonization can be accelerated at a more practical, sector‐ and country‐specific, level. We focus on CMs and UK/European policy to demonstrate the value of such a whole‐systems visualization and its potential to add value for other hard to decarbonize material cycles and geographic contexts.

## CEMENT

Cement is the key precursor of mortar and concrete, the latter being the most used synthetic material on Earth (IEA, 2018). It reacts with an activator (e.g., water) to form the relatively strong and durable matrix, known as a “binder,” that glues aggregate (sand, gravel) particles together in these materials. Cement is essential to socio‐economic development, through buildings, for homes, education, business, and healthcare, and other infrastructure, for transport, sanitation, and energy. Accordingly, global cement demand is expected to increase in the future, compounded by increasing population and urbanization. Global annual cement production almost tripled from 1994 to 2014 (USGS, 1996, 2015), and is expected to be 12–23% higher still by 2050 under a business‐as‐usual scenario (IEA, 2018). This increase in production is anticipated to come mainly from lower‐income countries. However, concrete will remain a key building material in higher‐income countries due to its relatively low production cost and abundant raw materials. Cement also facilitates decarbonization of other industries and end‐use sectors through use of concrete in construction, for example, wind turbines, dams, and buildings with low operational energy consumption. European cement production is expected to increase by up to ∼10% by 2050 (Material Economics, 2019).

Portland cement (PC)‐based concrete has a low embodied emissions intensity by mass (kg CO_2_‐eq./kg concrete) compared to other construction materials such as steel (Kua & Maghimai, 2017; Purnell, 2012). However, such CM products are used at such a massive scale that they account for ∼8% of total anthropogenic emissions and ∼23% of industrial emissions globally (Favier et al., 2019; Miller et al., 2016). PC clinker production accounts for most of the embodied emissions in PC concrete. It is one of the most difficult industrial processes to decarbonize, since fundamental “process emissions” account for ∼50% of PC production emissions (Davis et al., 2018; Teh, Wiedmann, Castel, & de Burgh, 2017).

### Cement decarbonization in the United Kingdom

In the United Kingdom, two thirds of industrial emissions arise from producing cement, ceramics, chemicals, food and drink, glass, iron and steel, oil refining, and pulp and paper (DECC, 2015a). In 2012, UK cement production accounted for ∼9% of UK industrial emissions (DECC, 2015b) and <1.5% of total UK emissions (MPA, 2015a; Shanks et al., 2019). From 1990 to 2015 the emissions intensity (kg CO_2_/unit product) of UK cement and concrete reduced by ∼27% and ∼22%, respectively (BEIS, 2017a; MPA, 2015a, 2015b). Environmental impacts other than those related to climate change arise from the CMs cycle, which should also be considered to avoid burden shifting. However, we limit our review to decarbonization due to the significant impact of the CMs cycle on climate change.

## CEMENT CYCLE

**FIGURE 1:**
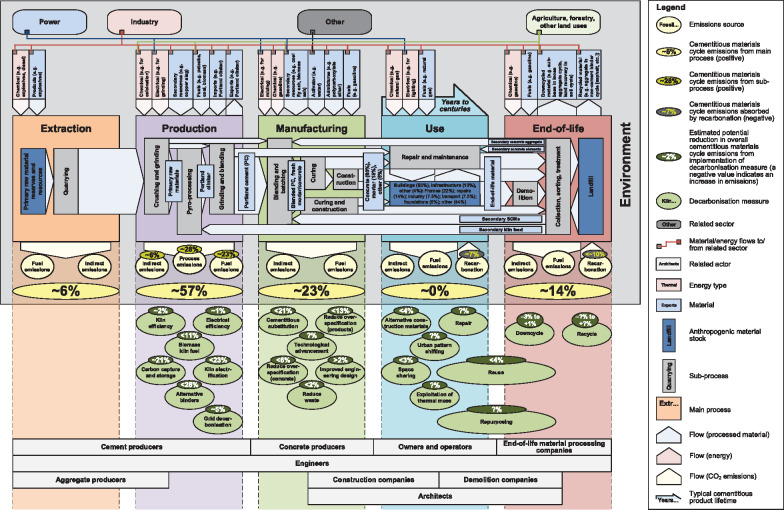
Cementitious materials (CMs) cycle annotated with some key aspects related to industrial decarbonization (as discussed in the text), drawn to represent the UK context. Colors are used for visual clarity only. The values presented refer to the average European cement in ca. year 2020, which contains ∼75% PC clinker and ∼25% non‐Portland CMs (NPCMs) (WBCSD, 2016). The underlying data used to create this figure are shown in Supporting Information S1

The cementitious materials (CMs) cycle consists of the following main processes (Figure 1): (1) extraction (of primary materials); (2) production (of cement and aggregate); (3) manufacturing (of concrete and mortar); (4) use; and (5) end‐of‐life. Calculations to estimate emissions from these processes (discussed below) are detailed in Supporting Information S2.

### Extraction

Limestone, clay, and shale are the three main raw materials used in PC clinker production. They are abundant, both globally and in the United Kingdom (BGS, 2014). Cement plants are generally located adjacent to raw material quarries to minimize transportation costs (BGS, 2014; van Oss & Padovani, 2002). Water and crushed rock, sand, and gravel, which are used as aggregates, are the main raw materials used in PC concrete manufacturing. Approximately 6% of CMs cycle emissions arise from extraction.

### Production

PC production involves crushing and grinding, pyro‐processing, and grinding and blending. During crushing and grinding, raw materials are milled and mixed in varying proportions, typically >80 mass% limestone and <20 mass% shale and/or clay. The ground mix is heated, or “pyro‐processed,” in a series of heat exchangers (“preheaters”) and a rotary kiln. At 600–800°C, the calcium carbonate (CaCO_3_) in limestone decomposes to calcium oxide (CaO) (calcination), emitting CO_2_ (process emissions). At ∼1450°C, PC clinker is formed. PC is produced by grinding and blending PC clinker with other reactive materials (in the context of cementitious systems), which modify its cementitious properties. We define these reactive materials as non‐Portland cementitious materials (NPCMs). At this step, two NPCMs are almost always used: limestone and a source of calcium sulfate, for example, gypsum, which is typically “inter‐ground”; ∼4 mass% of calcium sulfate is added to control setting of the cementitious binder (Hewlett, 2003). Other NPCMs may also be added in this step, or during batching (see Section 3.3). The location of their addition (i.e., at the cement or concrete plant) depends significantly on cost (e.g., transportation).

Our definition of an NPCM differs from a supplementary cementitious material (SCM) (Lothenbach, Scrivener, & Hooton, 2011). The term SCM is used to distinguish between the technical roles or properties of components in cement and excludes materials like gypsum (i.e., “solid admixtures”) that are used to modify specific properties of mortar or concrete, such as setting time. The term NPCM is used to simplify our discussion of cementitious substitution, the main objective of this measure being to reduce PC clinker content in a cement binder. Hence, we define an NPCM as any reactive material that contributes to the cementitious properties of a cement binder, excluding PC clinker. NPCMs include: non‐PC clinkers (e.g., calcium sulfoaluminate cement clinker); SCMs (e.g., coal fly ash), reactive fillers (e.g., fine limestone), solid admixtures (e.g., gypsum); and non‐water liquid and gaseous components such as SiO_2_ (aq) in a sodium silicate solution and CO_2_ (g) that readily become incorporated into the solid binder during curing (e.g., in alkali‐activated materials and through forced carbonation; see Section 3.3). It thus excludes: unreactive materials such as “inert fillers” (Berodier & Scrivener, 2014), which we rather classify as aggregate; and water.

For a typical blended PC with a clinker‐to‐cement ratio of 0.75, production accounts for ∼57% of CMs cycle emissions (Teh et al., 2017), which originate from three main sources: process emissions (∼28% of CMs cycle emissions), fuel (combustion) emissions (∼23% of CMs cycle emissions), and other emissions (∼6% of CMs cycle emissions) (Salas et al., 2016; Shanks et al., 2019). The greater two derive from pyro‐processing, while the other emissions are mainly associated with the electricity used by machinery to crush, grind, and blend raw materials and PC clinker.

### Manufacturing

Manufacturing includes batching, curing, and construction. Batching refers to the mixing of CMs (e.g., PC clinker, NPCMs), aggregates, and an activator (e.g., water in PC products). Most NPCMs are added (to produce the desired PC type) during concrete batching in the United Kingdom (DECC, 2015b; MPA, 2015a). Common NPCMs include blast furnace slag, coal fly ash, limestone, and gypsum. Aggregates vary in size depending on the required product. Mortar uses fine aggregates (e.g., sand), while concrete uses fine and coarse aggregates (e.g., rock, gravel). Alternative activators to water are used in non‐PC binders such as alkali‐activated materials; they are used to accelerate setting and may include NPCM components (e.g., SiO_2_(aq)). Alternative activators may be liquid (e.g., aqueous NaOH solution), solid (e.g., solid NaOH), or both (e.g., water and solid NaOH can be used in “one‐part” alkali‐activated materials). Liquid activators are typically an aqueous solution added at the batching stage, and their addition, similar to water, produces a “wet” batching product. Gaseous activators also exist, for example, CO_2,_ and are added during curing. Once CMs are mixed with an activator, the mixture reacts to form a “binder,” which eventually sets, binding the aggregates together. Curing refers to the process by which this reaction is controlled (using temperature and humidity), to provide good material property development.

There are five main (blended) PC types specified in Europe under ISO EN 197‐1 (BSI, 2011): CEM I–V. Their constituents are quantified in mass% excluding calcium sulfate additions (typically gypsum). CEM I (ordinary PC, OPC) contains the highest proportion (95–100 mass%) of PC clinker. The remaining 0–5 mass% (“minor additional constituents” in ISO EN 197‐1) is typically limestone. CEM II–V are commonly referred to as blended PCs or “composite” cements: they have PC clinker contents ranging from 5−94 mass% (BSI, 2011). On average, PC clinker comprises ∼75 mass% of European cement (WBCSD, 2016). Here, we use the term PC to refer to “ordinary PC” as well as “blended PC” for simplicity, that is, it refers to mixtures of PC clinker and NPCMs, which thus includes essentially all cements used today (in terms of quantities used).

PC concrete has three main sales routes: ready‐mix, pre‐cast, and retail (55, 25, and 15 mass% of UK cement use, respectively; Shanks et al., 2019). Ready‐mix is produced at wet batching plants and sold at scale. The wet batching product is transported by truck to the construction site where it is cured on site during construction. In pre‐cast manufacturing, components are cured in fit‐for‐purpose factories. Wet batching product is poured into a reusable mold and cured. Once set, the component is removed from its mold and transported to the construction site. Retail, like ready‐mix, is cured on site. It is sold on a much smaller scale in the United Kingdom, as “dry” batching product (without water), in bags.

The PC clinker intensity in concrete is ∼0.1 kg PC clinker/kg concrete in Europe (European Commission, 2014; Favier et al., 2018), and the binder intensity in concrete is usually ∼350 kg binder/m^3^ concrete internationally (Damineli, Kemeid, Aguiar, & John, 2010). PC clinker intensity shows a strong positive correlation with embodied emissions (i.e., concretes with higher PC clinker intensity have higher embodied emissions), and its value depends on various factors, including specified performance, for example, compressive strength. Durability is one such common performance characteristic, which is represented in terms of “exposure classes,” and includes resistance of (reinforced) concrete to: corrosion, chemicals (e.g., sulfates), moisture, and temperature (BSI, 2000).

Manufacturing emissions comprise ∼23% of CMs cycle emissions (Teh et al., 2017). Notable contributions to this total are from electricity use (∼5% of CMs cycle emissions) and road transport (∼2% of CMs cycle emissions).

### Use

In the United Kingdom, PC is predominantly used in buildings (83 mass%) and infrastructure (13 mass%) (Shanks et al., 2019). In buildings, PC is used in structural frames (22 mass% of PC demand), including columns, flooring, walls, and beams; in foundations (5 mass% of PC demand); and in industry (7.5 mass% of PC demand) (Shanks et al., 2019). In infrastructure, PC uses include tunnels, pipes, pavement, and transport (7.5 mass% of PC demand), for example, railways and bridges. Repairs and maintenance of such products account for 14 mass% of PC demand (Shanks et al., 2019). Concrete can be reinforced (containing steel bars to improve tensile strength) and unreinforced, depending on its end‐use. Reinforced concrete generally requires a more reliably durable exposure class. In Europe, reinforced concrete accounts for 75% of all concrete produced, including ∼90% of ready‐mix concrete and ∼50% of pre‐cast concrete (data are for year 2015) (Favier et al., 2018).

Building and infrastructure emissions comprise two components: operational, related to using the product, including lighting and heating/cooling, and embodied, related to provision of the product, including construction, maintenance, and demolition. Operational emissions are usually larger than embodied emissions in many buildings but are decreasing with increasing deployment of renewable energy and energy efficiency measures. Embodied emissions are generally considered dominant in infrastructure, although high operational emissions cases of >50% exist (Kirchain, R. E., Gregory, & Olivetti, 2017). In the United Kingdom, embodied emissions range from 3–80% of total buildings and infrastructure emissions (Ibn‐Mohammed, Greenough, Taylor, Ozawa‐Meida, & Acquaye, 2013), although can be >60% for typical modern offices, warehouses, and residential buildings (Royal Institute of British Architects, 2017).

### End‐of‐life

Buildings and infrastructure often reach end‐of‐life before the mortar and concrete used in them degrade. Following their use, they are demolished or deconstructed into mortar and concrete fragments of varying size, which are then collected at sorting plants. These materials are classified as secondary coarse and fine aggregates, which may be: processed and recycled in the CMs cycle or other material cycles (e.g., asphalt); downcycled (e.g., “loose” use of secondary aggregate with poorer qualities than virgin aggregate as road sub‐base); or disposed of in landfill. In Europe downcycling end‐of‐life cementitious products into road sub‐base is the predominant mode of treatment.

Most LCA studies of cementitious products have cradle‐to‐gate scopes that omit end‐of‐life treatment, in part due to poor data availability (Laurent et al., 2014; Salas et al., 2016; Teh et al., 2017; Vieira, Calmon, & Coelho, 2016). End‐of‐life processes (excluding recarbonation) are cumulatively estimated to account for ∼14% of CMs cycle emissions in Europe (De Schepper, Van den Heede, Van Driessche, & De Belie, 2014).

## Decarbonization measures

The landscape of the cement decarbonization literature is diverse in terms of scope (e.g., measures, system boundaries), method (e.g., LCA, back‐of‐the‐envelope calculation), assumptions, and reproducibility (i.e., (in)completeness of the description of the underlying data and methods). This makes it challenging to assess their relative importance using a singular consistent method. Therefore, we describe our estimates of decarbonization potentials (Figure 1) as semi‐quantitative, cautiously discuss them on a case‐by‐case basis (in Section 4 and subsections), and summarize the sources and calculations used to develop them in the Supporting Information S1 and S2.

### Energy efficiency

The decarbonization potential of energy efficiency in industry is limited due to historical improvements implemented over many years (Favier et al., 2018). Since pyro‐processing is the most energy intensive process in PC production, the cement kiln is the focus of most energy efficiency improvements. The most important energy efficiency measure for cement kilns is upgrading from wet to dry processing. However, dry processing is already used in 86% of global PC clinker production (Shanks et al., 2019). Other measures include use of preheaters, pre‐calciners, oxygen enrichment, and by‐product heat. Kiln‐based energy efficiency measures are thus estimated to be able to further reduce UK CMs cycle emissions by ∼2% (DECC, 2015b) (Figure 1).

Electrical efficiency measures, including voltage and power optimization, and use of improved equipment, can reduce costs for cement plants and CMs cycle emissions by ∼1 % (Figure 1) (DECC, 2015b).

Concrete can enhance energy efficiency when used in buildings, by reducing operational heating (by ∼ <20%) and/or cooling (by ∼ <5%) requirements through its thermal mass (i.e., its heat capacity) (Hacker, De Saulles, Minson, & Holmes, 2008). This practical utility of concrete as a means of energy storage is symbiotic with (fluctuating) renewable energy supply, and so can support higher penetration of renewable energy, leading to overall emissions reductions. Although it has been estimated that this may lead to up to 25% emissions reductions per structure (3E, 2016), the complex interplay among electricity generation and storage, material selection, design, and operation of buildings makes it challenging to assign a potential emissions reduction to this use of CM products. Therefore, a value has not been assigned to this decarbonization measure (“exploitation of thermal mass”) in Figure 1.

### Emissions efficiency

Emissions efficiency measures focus mainly on process and fuel combustion emissions from pyro‐processing, which are the main sources of CMs cycle emissions (∼28% and ∼23%, respectively). Reducing these emissions per unit of cement produced will require a fundamental change to cement production in terms of energy source, equipment, or raw materials.

#### Carbon capture and storage

Most cement decarbonization scenarios include carbon capture and storage (CCS) implemented at PC clinker production. Here, various CCS technologies are possible (Boot‐Handford et al., 2014). However, a key distinction is whether they capture CO_2_ from the combined process and fuel emission streams (“combined CCS”), or from separate process and fuel emission streams (“direct CCS”). Process emissions contain essentially pure CO_2_ originating from limestone calcination (CaCO_3_ → CaO + CO_2_), whereas fuel emissions contain CO_2_ and other gases, for example, N_2_ if air is used as the combustion gas.

Combined CCS separates CO_2_ from mixed process‐fuel emissions streams by using technologies such as amine scrubbing and calcium looping. Other combined CCS technologies act to reduce the concentration of non‐CO_2_ gases in this mixed stream, for example, oxyfuel combustion, reducing the complexity of subsequent CO_2_ separation. Direct CCS technologies capture pure CO_2_ (e.g., from the preheater/precalciner) by physically separating the limestone calcination reaction from the kiln fuel (Hills, Leeson, Florin, & Fennell, 2016; LEILAC, 2020). Some technologies can achieve either combined or direct CCS depending on their installed configuration. For example, in pre‐combustion CCS (Leung, Caramanna, & Maroto‐Valer, 2014), H_2_ and CO_2_ are produced, separated, and then the H_2_ can be used as a kiln fuel with no direct GHG emissions. Directly heating the kiln using H_2_ as fuel, that is, with no physical separation between fuel burning and limestone calcination, is compatible with combined CCS. Whereas, indirect heating using H_2_ as fuel, for example, by heating a vessel wall that physically separates fuel burning from limestone calcination, is compatible with direct CCS. Use of H_2_ produced by electrolysis is also compatible with either combined or direct CCS. Kiln electrification technologies, for example, plasma and microwave, are compatible with direct CCS. The literature suggests that oxyfuel type combined CCS technology is preferred for implementation in PC clinker production (DECC, 2015b; Material Economics, 2019).

A major barrier to implementation of CCS is its relatively high cost (Liang & Li, 2012). Carbon capture and use (CCU) may improve the economic viability of CCS by providing a revenue stream through CCU products, independent of carbon trading markets. However, the demand for CCU products would need to be high—comparable to PC clinker production quantities—to significantly affect CMs cycle emissions (Favier et al., 2018). Additionally, these products would need to have comparable lifetimes to cementitious products to act as a significant carbon sink, casting doubt on the CMs cycle decarbonization potential of CCU chemical products (Cuéllar‐Franca & Azapagic, 2015). A potentially suitable CCU product may be synthetic aggregates (Schneider, 2020). Therefore, the potential of CCU to reduce CMs cycle emissions is uncertain, and no decarbonization potential has been estimated for it here.

CCS is a relatively immature technology in the cement industry, with only one active demonstration plant in the EU (Bjerge & Brevik, 2014; Material Economics, 2019). In addition to technical and economic challenges, CCS faces logistical issues due to the geographically dispersed nature of cement plants that may or may not be nearby suitable CO_2_ storage locations. Despite these issues, CCS is commonly projected to have a high potential to reduce CMs cycle emissions. Here, we estimate its CMs cycle decarbonization potential to be ∼21% (DECC, 2015b), which is consistent with a 50% application rate of CCS to cement production, is in line with other published decarbonization roadmaps (Material Economics, 2019), and requires the aforementioned challenges related to implementation of CCS technology to be at least partially overcome.

#### Enhanced recarbonation

Cement binders “recarbonate” by absorbing atmospheric CO_2_, potentially capturing ∼7% CMs cycle emissions during use (Cao et al., 2020; MPA, 2013; Pade & Guimaraes, 2007). This process is recognized (e.g., by the IPCC) but is not included in national emissions inventories as of 2020 (CEMBUREAU, 2020). Recarbonation during use may be increased through targeted structural design.

The CO_2_ absorbed by mortar and concrete during end‐of‐life depends on how these materials are processed, ranging from ∼1% CMs cycle emissions if landfilled, to ∼10% if crushed and downcycled as sub‐base (Pade & Guimaraes, 2007 ). Therefore, CMs cycle emissions can be reduced by optimizing end‐of‐life processing to enhance recarbonation. We show the upper bound of the decarbonization potential for recarbonation in the end‐of‐life stage (10%) in Figure 1.

#### Alternative energy sources

Conventional fossil fuels, for example, coal and petcoke, made up 54% of thermal energy use in pyro‐processing in Europe in 2017 (GCCA, 2017). Cement kiln development has historically centered around using solid fuels (Akhtar, Ervin, Raza, & Abbas, 2016). Hence, research into and implementation of alternative energy sources in cement production has focused on alternative solid fuels such as secondary fuels and biomass. Literature on liquid and gaseous energy sources is much sparser. Therefore, given their limited decarbonization potential relative to biomass and electrification, we omit oil and natural gas from this review.

##### Secondary fuels and biomass

Use of secondary fuels (e.g., tires, end‐of‐life plastics) in the United Kingdom almost doubled from 2008 to 2015, with similar uptake in Europe. However, accounting for their emissions is complex since the energy content of many secondary fuels derives from fossil carbon. Their decarbonization potentials depend on the way this fossil carbon is allocated, as well as their emissions intensities when burned during pyro‐processing, which is lower than conventional coal and petcoke (Habert, Billard, Rossi, Chen, & Roussel, 2010). The same is true for biomass fuels. Due to the already significant use of secondary fuels in Europe (∼20% in 2014 (IEA, 2018)), and the arguably greater potential of biomass fuels to reduce emissions, we focus on quantifying the latter here.

Biomass makes up 14% of thermal energy use in pyro‐processing in Europe (GCCA, 2017). Increased uptake is restricted by land availability, which is under competition from numerous other end‐uses (e.g., food production). Biomass also faces technical limitations like requirements for additional treatment due to its lower calorific value than conventional fuels and potential impacts on PC clinker chemistry (Favier et al., 2018). Ignoring supply constraints, use of biomass as fuel could reduce UK CMs cycle emissions by ∼11% from 2012 to 2050 (DECC, 2015b). This is a highly optimistic value that should be interpreted with caution; supply constraints may also limit their decarbonization potential to a negligible value (Material Economics, 2019). It is important to note here that cement plants can beneficially utilize low‐grade biomass such as agricultural residues as a minor component of the kiln fuel mix (Rahman, Rasul, Khan, & Sharma, 2015). Since these materials may have limited other applications, use of biomass fuels provides waste treatment benefits beyond the CMs cycle.

##### Electrification

Electrification of the cement kiln, directly or indirectly (e.g., by generating H_2_) may significantly change the PC clinker production process, since it was developed for solid fuels. Therefore, electrification may require substantial investment, expense, and disruption to PC clinker production; there are also concerns over its effects on product quality and kiln performance, and its suitability to be applied at temperatures above ∼1000°C that are required to produce PC clinker. However, electrification has the potential to eliminate direct fuel combustion emissions (∼23% of CMs cycle emissions, Figure 1), and with low enough electricity prices (<EUR 40/MWh), make direct capture CCS comparable in cost to combined CCS (Material Economics, 2019). Therefore, it has among the highest potential out of all decarbonization measures. Assuming a net‐zero source of electricity the following electrification measures could reduce CMs cycle emissions by <∼23%.

##### Hydrogen

Hydrogen may be used to provide heat directly or indirectly during pyro‐processing (Section 4.2.1). Indirect heating would require substantial modifications to cement plants (Material Economics, 2019). Direct heating is not discussed in the reviewed literature other than as an implied component of pre‐combustion CCS or in combination with secondary fuels and oxy‐fuel combustion (Farfan, Fasihi, & Breyer, 2019; Material Economics, 2019). Hydrogen has the potential to enable further indirect emissions savings, for example, its production during electrolysis of O_2_ for both oxyfuel CCS and increased demand flexibility in the power system (Bataille et al., 2018; Lechtenböhmer, Nilsson, Åhman, & Schneider, 2016).

##### Plasma and microwave

Plasma generators have been proven in industrial contexts and can generate the required temperatures for some cement production steps with an efficiency of 85–90% (Material Economics, 2019). Microwave technology has not yet been used at scale in industrial processes; however, it is routinely used at lower temperatures and has the potential to increase the energy efficiency of cement kiln heating by <40% (Material Economics, 2019).

#### Grid decarbonization

While indirect emissions from electricity supply comprised ∼5% of CMs cycle emissions in 2019, many decarbonization measures require greater electrical power demand. Therefore, decarbonization of the power sector is needed to prevent increased indirect emissions from electricity supply, highlighting the importance of taking a whole‐systems approach (i.e., accounting for interdependencies among different industries) in decarbonization of the CMs cycle. Decarbonization of the UK electricity grid has the potential to reduce CMs cycle emissions by ∼5% (DECC, 2015b).

### Material efficiency

**FIGURE 2:**
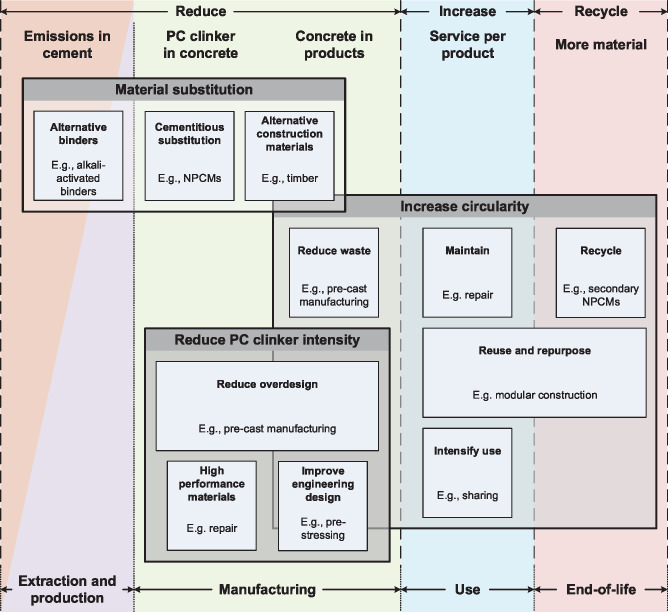
Material efficiency measures and their locations in the cementitious materials (CMs) cycle. NPCMs are non‐Portland cementitious materials

Material efficiency is key to facilitating industrial decarbonization by reducing the rate, scale, and cost of deploying energy and emissions efficiency measures. Material efficiency measures span the entire CMs cycle and can be differentiated by type, technology, target material, actor, etc. (Figure 1). Here, we categorize them, firstly by type (material substitution, reduce PC clinker intensity, and increase circularity), and secondly by their locations in the CMs cycle (Figure 2). We include this latter categorization to maintain a consistent systems perspective. We define these locations as follows (1–5):

1. Reduce emissions in cement, for example, replacing PC binders with alternative binders that have lower cradle‐to‐gate CO_2_ emissions;
2. Reduce PC clinker in concrete, for example, reducing overspecification and using NPCMs;
3. Reduce concrete in products, for example, reducing overspecification and increasing use of technology like 3D printing, pre‐cast, and post‐tensioning;
4. Increase service per product, for example, extending lifetimes of buildings and their components through reuse and refurbishment;
5. Increase recycling, for example, processing of construction and demolition waste concrete into NPCMs rather than aggregates.

Cumulatively, it is thought that material efficiency measures have the potential to reduce CMs cycle emissions by <∼38% (Favier et al., 2018; Material Economics, 2019; Shanks et al., 2019).

#### Material substitution

Material substitution describes the substitution (partial or complete) of one material for another with similar functionality. Such a substitution may or may not lead to a reduction in environmental impact. Here, we classify material substitutions into three main categories (1–3):

1. Alternative binders. PC binder (comprising PC clinker, NPCMs, and water) can be substituted by different combinations of CMs and/or activators. For example, calcium sulfoaluminate clinker, NPCMs, and water (Hanein, Galvez‐Martos, & Bannerman, 2018); and carbonatable calcium silicate clinker, NPCMs, and CO_2_ (Miller & Myers, 2020).
2. Cementitious substitution. PC clinker and PC can be substituted by NPCMs, for example, coal fly ash.
3. Alternative construction materials. Mortar and concrete can be substituted by different materials, for example, timber (Churkina et al., 2020).

##### Alternative binders

Alternative binders may have (potentially much) lower embodied emissions than PC binder per unit mass due to their different chemistries, raw materials, and properties (Habert & Ouellet‐Plamondon, 2016; Teh et al., 2017). They include alkali‐activated binders, which are mixtures of aluminosilicate precursors and usually an alkaline aqueous solution (as the activator), and sometimes referred to as geopolymers (Provis, 2018). Alternative binders also include use of non‐PC clinkers and cements, such as belite‐ye’elimite‐ferrite and carbonatable mixtures of calcium and/or magnesium (hydr)oxides (Gartner & Sui, 2018; Miller & Myers, 2020). However, their potentials to reduce emissions, technological maturities, and “technical performance” (e.g., durability, setting times, compressive strength) vary widely, limiting their application. Non‐reinforced concretes generally have higher potentials for alternative binders and cementitious substitution, since their risks and consequences of failure during service is lower: steel corrosion in reinforced concrete is one of the most important durability issues. Substitution of alternative binders for conventional blended PC binder could reduce UK CMs cycle emissions by < ∼28%. This value was determined recognizing the prevailing ∼25% rate of PC clinker substitution in Europe (in 2018) (WBCSD, 2016)(GCCA, 2017). We consider this to be an optimistic value, since it assumes an unrestricted supply of their raw materials, which is unlikely to be the case (e.g., for blast furnace slag, which is generated at ∼10% of the rate of PC production (USGS, 2020).

##### Cementitious substitution

Using our definition of non‐Portland cementitious materials (NPCMs) introduced in Section 3.2, five main NPCM types are used today: calcium sulfate (e.g., gypsum), limestone, primary natural pozzolans (e.g., volcanic ash), primary synthetic pozzolans (e.g., calcined clay), and secondary materials (e.g., coal fly ash, a coal combustion by‐product, and blast furnace slag, a by‐product of pig iron production). Gypsum, limestone, and secondary materials (coal fly ash, blast furnace slag) are currently the most used NPCMs in the United Kingdom, with the latter accounting for 20% of CMs used (DECC, 2015b; Shanks et al., 2019). Finely ground limestone is widely used in Europe and globally. As mentioned above, the average substitution rate of PC clinker (over all PC types, CEM I‐V) is 25% in Europe (in 2018) (GCCA, 2017). The most commonly used NPCMs are generally allocated a much smaller fraction of embodied CO_2_ emissions than PC clinker (Habert & Ouellet‐Plamondon, 2016). Therefore, CMs cycle emissions can be reduced by increasing the cementitious substitution rate, i.e., decreasing the average PC clinker content in cement.

The cementitious substitution average is limited significantly by NPCM availability and reactivity (which importantly contributes to early compressive strength development). The cementitious substitution average in Europe could increase to up to 50% due to, for example, the technological maturity and good technical performance of limestone‐calcined clay‐cement (LC^3^) (Scrivener, Martirena, Bishnoi, & Maity, 2018). This cementitious substitution average of 50% corresponds to a potential reduction in UK CMs cycle emissions of <∼21%.

##### Alternative construction materials

In some applications, for example, structural elements in buildings, concrete can be substituted for alternative materials. Concrete has relatively low embodied emissions per unit volume and mass compared to timber and steel (Purnell, 2012). However, much larger masses of concrete than timber are required to construct equivalent products (Hertwich et al., 2019). The lower embodied emissions of timber products often outweigh the higher operational emissions arising from their lower thermal masses compared to concrete products (Heeren et al., 2015; Hertwich et al., 2019).

Increasing timber construction is controversial due to the challenge of guaranteeing its environmentally sustainable sourcing and competing demands for both timber and forest land area, especially at the scale required for significant concrete substitution. Timber is currently used in lower quantities than concrete (timber represents ∼3 mass% of all construction materials used) (Schandl et al., 2016), meaning that a massive expansion in supply would be needed to facilitate moderate timber‐for‐concrete substitution rates (Oliver, Nassar, Lippke, & McCarter, 2014). Safety and performance codes may further restrict substitution of concrete for timber, and, like novel concrete materials, standards may not necessarily support technical advancements in timber, for example, engineered wood products. Relatively few scenarios have included concrete for wood substitution due to these complexities. Where it has been included, usually a conservative substitution extent is assumed, for example, 5 mass%, or marginal reductions in emissions by 2050 are found (Göswein, Krones, Celentano, Fernández, & Habert, 2018; Heeren & Hellweg, 2019; Material Economics, 2019). Very ambitious scenarios, both in terms of land and forest management, and timber for concrete substitution, are required to substantially increase this value (Churkina et al., 2020). We thus estimate a <4% reduction in CMs cycle emissions from use of alternative construction materials here.

#### Reduce PC clinker intensity

##### Reduce overdesign

Commonly, more PC (clinker) is used than necessary to meet the specified performance of a component in a product with respect to engineering standards. This overdesign is driven mainly by “safety factors” and logistical optimization. Safety factors are applied by engineers to reduce the risk of poor technical performance, for example, from potentially suboptimal manufacturing conditions at construction sites, and typically correspond to +20% in material use (Favier et al., 2018; Material Economics, 2019; Moynihan & Allwood, 2014; Passer, Deutsch, & Scherz, 2018). Logistical optimization aims to save time and reduce complexity of construction by using standardized product designs and concrete classes for potentially all components, rather than generating project specific designs and assigning specific concrete exposure classes to each component. These practices are common in building construction, where ease and speed of construction is prioritized. Logistical optimization results in more PC clinker, cement, mortar, and concrete being used than technically necessary.

Reduced overdesign can be achieved through various measures: increased digitization in construction, for example, additive manufacturing; better matching of materials or material properties and products, for example, concrete exposure classes and concrete components; better tracking of cement use and intensity (Favier et al., 2018); increased use of pre‐cast over ready‐mix concrete; and use of planning systems to optimize cost, constructability, and embodied emissions, for example, building information modeling. In the United Kingdom, reducing overdesign is estimated to have the potential to reduce CMs cycle emissions by <∼6% for PC in concrete, and <∼13% for concrete in products (Shanks et al., 2019).

##### High‐performance materials

High‐performance materials reduce PC clinker intensity in cement products by improving properties of cement products at constant PC clinker content. Technologies include:

- Optimizing particle size distribution of solids in fresh mortar and concrete, which may be achieved through advancements in grinding, particle dispersants (i.e., chemical admixtures), high‐quality aggregates, and use of fillers (John, Damineli, Quattrone, & Pileggi, 2018).
- Using strength accelerators to improve early age compressive strength of cementitious binders, making lower PC clinker content cements suitable for more applications.
- Improving durability (e.g., increased resistance of concrete to chlorine‐ and sulfur‐induced degradation) to facilitate other material efficiency measures such as more intense product use, reuse, and NPCM use.

An estimate of the cumulative decarbonization potential of all such measures has not been determined due to the complexity of each individual measure and their interconnected nature. However, one such performance improvement, the injection of CO_2_ during ready‐mix batching and curing as a strength accelerator, is claimed to reduce CMs cycle emissions by <∼4% (Monkman & MacDonald, 2017).

##### Improved engineering design

The PC clinker intensity of cementitious products can be reduced by improving the structural design of components to deliver the same performance with less concrete. For example, pre‐stressing or post‐tensioning of steel reinforcement (to provide tensile strength to the concrete during use), and partially hollow/optimized structural elements. Pre‐cast manufacturing can facilitate such measures by providing improved precision over ready‐mix manufacturing (Favier et al., 2018). We estimate the decarbonization potential of one such improved engineering design measure rather than an estimate of the cumulative potential due to the high number of possible improvements: post‐tensioning in reinforced concrete is estimated to have a CMs cycle decarbonization potential of <∼2% (Shanks et al., 2019).

#### Increase circularity

Circular economy is a concept that views economic activities as service focused “circular” systems, rather than production focused “linear” systems (Korhonen, Honkasalo, & Seppälä, 2018). The concept builds on decades of sustainability science research, for example, in industrial ecology (Bocken, Olivetti, Cullen, Potting, & Lifset, 2017). Circular systems have the potential to improve environmental sustainability (Cullen, 2017) and economic value particularly for actors involved in service provision (World Economic Forum et al., 2016). They notably involve strategies to retain products and materials in use, by maintaining, reusing, intensifying use, and recycling.

##### Reduce waste

Significant amounts of secondary materials (‘waste’) are generated during concrete manufacturing and demolition. Waste reduction measures include improving construction practices and using advanced construction techniques, for example, additive and pre‐cast rather than ready‐mix manufacturing (Reis, Mack‐Vergara, & John, 2019). Such waste reduction measures have been estimated to reduce CMs cycle emissions in Europe by <∼1% (Material Economics, 2018; Shanks et al., 2019). However, it is unclear which technologies are included in such estimates. We estimate the decarbonization potential of one such technology that reduces ready‐mix batch reject rates through optimizing water and admixture dosage rates to be <∼2% (Myers 2020, personal communication). Therefore, we expect the cumulative potential of reducing waste to be significantly higher.

##### Maintain and reuse

Buildings are often demolished for aesthetic and economic rather than technical performance reasons (e.g., structural degradation). Designing and using modular structures that can be extracted and reused in the same, or another building facilitates repair, refurbishment, repurposing, and reuse of buildings and components rather than their demolition.

Repairs and maintenance comprised 14% of UK cement production in 2015 (Shanks et al., 2019). However, the exact cement containing products being repaired were not reported. More specific data on cement use in products is needed in order to analyze the extent to which repairs may contribute to improved material efficiency through increased lifetimes of products. Since it is easier to replace modular components, repairing of products can be facilitated by increasing use of pre‐cast concrete and modular structural design. We did not estimate the decarbonization potential of this measure.

Few studies of component reuse that have been implemented in practice exist (Arora, Raspall, Cheah, & Silva, 2019). Uptake of component reuse is limited by supply: few modular components have been used historically; therefore, matching specifications of supplied with demanded components (in the right place and time) is complex. Component connections also often require modification (remanufacturing) (Favier et al., 2018). Digitization may help overcome these limitations by clarifying prevailing supply and demand for such components, as well as their properties/specifications. Increasing reuse of components has been estimated to have a potential to reduce CMs cycle emissions in Europe by <∼4% by 2050 (Material Economics, 2019). The CMs cycle decarbonization potential of repurposing structures, which we consider to be a special case of reuse involving potentially many components, has not been estimated here.

Although technical measures like pre‐cast and additive manufacturing (e.g., 3D printing) have relatively low decarbonization potentials now, increased demand for modular components could fundamentally change construction. This may eventually result in much larger CMs cycle emissions reductions in the future. For example, pre‐cast manufacturing shifts assessment of performance from the material level (e.g., cement binder in EN 197‐1) to the component level. This will enable other material efficiency measures with higher decarbonization potentials, such as use of unconventional materials in material substitution cases. Therefore, the decarbonization potentials reported here for pre‐cast and additive manufacturing should be treated as lower bounds, and the decarbonization potentials of technological advancements (e.g., new materials) are unclear yet possibly high.

##### Intensify use

Intensifying product use reduces the amount of product needed to provide a service. This can be facilitated through thoughtful product design to provide multiple functions without significantly increasing material use, potentially avoiding the need for multiple products, and sharing centric business models (e.g., Airbnb Inc.). An example of multi‐function product design is permeable concrete (Kia, Wong, & Cheeseman, 2019), which may reduce the need for separate drainage products in infrastructure systems. More intense use from space sharing is estimated to reduce CMs cycle emissions in Europe by <∼3% by 2050 (Material Economics, 2019).

##### Recycling

**FIGURE 3:**
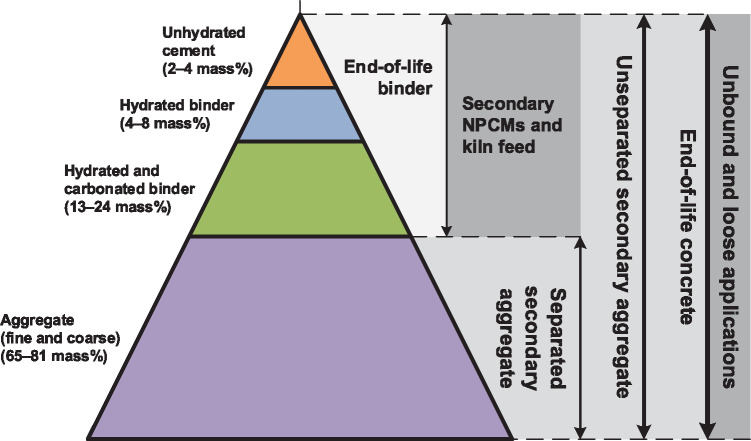
Classification of end‐of‐life concrete and some reuse cases. The average values in the percentage ranges on the left hand side of the figure correspond to areas shown in the triangle. Higher value uses of end‐of‐life concrete are shown toward the top of the triangle. NPCMs are non‐Portland cementitious materials

Recycling improves material efficiency through substitution of primary for secondary materials. Here, we consider a range of processing routes, spanning recycling to “higher value” materials like NPCMs, to “lower value” materials, for example, aggregates. Downcycling refers to the use of any secondary material for an application of less value than its original purpose (Allwood, 2014; Graedel et al., 2011) (Figure 3).

In the United Kingdom, most end‐of‐life concrete is downcycled into secondary aggregate for loose applications (i.e., non‐concrete) below ground, for example, road sub‐base (Engelsen, Mehus, Pade, & Sæther, 2005; MPA, 2015a), but to a minor extent is also landfilled. We classify these as “open‐loop” forms of downcycling since CM is being used in loose rather than bound applications. “Closed‐loop” downcycling of end‐of‐life CM into mortar and concrete (i.e., bound applications) is also possible. Here, aggregates may or may not be separated from the binder. Unseparated secondary aggregates include both aggregates and binder. They have lower compressive strengths than primary and separated secondary aggregates. Therefore, concrete manufactured using unseparated secondary aggregates usually requires a higher PC clinker intensity to achieve the same compressive strength relative to concrete containing primary or separated secondary aggregates. Consequently, the decarbonization potential of downcycling end‐of‐life concrete into unseparated secondary aggregates is often negligible and sometimes negative, and here is estimated as to have a decarbonization potential +1 to −3% (i.e., an increase in overall CMs cycle emissions) (Yazdanbakhsh, Bank, Baez, & Wernick, 2018). This range is sensitive to the transport distances between the concrete plant, primary aggregate quarry, and secondary aggregate treatment facility. Use of net‐zero emission transport in downcycling of unseparated secondary aggregate may thus only lead to marginal reductions in CMs cycle emissions.

Technologies to recycle separated course and fine (sand) aggregates for use in mortar and concrete have been studied; however, we were unable to reliably estimate their decarbonization potentials. The other fraction of separated end‐of‐life concrete, end‐of‐life binder “fines” (the fine particles generated once the binder is separated from the aggregates), can be recycled if used as secondary PC (clinker) and/or NPCMs, or downcycled if used as PC kiln feed. These materials may be generated by:

1. Separating unhydrated, hydrated, and/or carbonated PC (clinker) fractions from end‐of‐life PC binder (Bordy, Younsi, Aggoun, & Fiorio, 2017; Lothenbach & Winnefeld, 2006; Oksri‐Nelfia, Mahieux, Amiri, Turcry, & Lux, 2016). The former material may then be recycled as PC (clinker), and the latter two materials may be recycled as NPCMs.
2. Calcining end‐of‐life PC binder to produce a clinker, potentially with similar chemistry to conventional PC clinker (Florea, Ning, & Brouwers, 2014), as a form of downcycling.

End‐of‐life PC binder can substitute PC clinker at similar levels to other NPCMs, depending on treatment method (Bordy et al., 2017; Florea et al., 2014). Use of untreated end‐of‐life binder in a cement kiln has produced a clinker with a mineralogical composition close to PC clinker and with ∼33% less process emissions from pyro‐processing (Gastaldi et al., 2015). However, technological improvements are generally required to enhance the quality of secondary NPCMs and kiln feed (i.e., to reduce contamination) produced from end‐of‐life CMs (Bakker et al., 2015; Lofti & Rem, 2016). Additionally, implementing these measures at the industrial scale requires changes to demolition practices, including increased collection rates, and improved separation of construction and demolition waste into distinct material types (Material Economics, 2019). Standards can be updated to promote use of end‐of‐life mortar and concrete in PC production and concrete manufacturing. Modular construction and other techniques promoting “design for disassembly,” including ‘material passports’, can enhance sorting of end‐of‐life CMs.

Concrete containing limestone aggregate that can directly be used as secondary cement kiln feed (i.e., without an aggregate/binder separation step) has also been designed (De Schepper et al., 2014). However, inconsistent application of allocation in that study prevents a reliable assessment of the decarbonization potential of this technology.

It is not yet clear whether re/downcycling concrete into secondary NPCMs and kiln feed would have a net negative or positive effect on CMs cycle emissions. Therefore, we do not provide an estimate of the decarbonization potential of this measure.

### Policy progress and projections

Momentum has been building for increased UK policy support in industrial decarbonization. Since the publication of the “Industrial Decarbonisation & Energy Efficiency Roadmaps to 2050” report in 2015 (DECC, 2015a), several initiatives have been launched that support implementation of industrial decarbonization measures beyond energy efficiency (BEIS, 2019). This includes recent policy strategies published by the UK government (DEFRA, 2018, 2020), which focus on improving material efficiency and reducing material losses related to many products used by industry, households, and in the commercial sector. However, policy initiatives for material efficiency measures and non‐CCS emissions efficiency measures in the CMs cycle are less well developed (BEIS, 2017a, 2019; DECC, 2015b). In general, a similar situation exists in Europe (Material Economics, 2019). Therefore, decarbonization of the UK and European CMs cycles presently relies largely upon actions undertaken by cement producers, who can only achieve net‐zero emissions through large‐scale deployment of CCS. Figure 1 demonstrates the range of other policy foci that can be explored to facilitate net‐zero CMs cycle emissions, including support for reducing overdesign and increasing recycling and recarbonation of end‐of‐life CMs.

Under a business‐as‐usual scenario, European cement production emissions are expected to remain stable until 2050: reductions in cement production emissions from energy efficiency improvements and power sector decarbonization measures are expected to balance an increase in emissions arising from a 10% increase in cement production (Material Economics, 2019). In the UK context, a business‐as‐usual scenario is expected to decrease cement production emissions by ∼12% from 2012 levels by 2050: UK cement production volumes are expected to remain stable, with emissions reductions originating from power decarbonization measures (DECC, 2015b). Evidently, business‐as‐usual practice will not lead to significant reductions in CMs cycle emissions.

Several roadmaps applying ambitious levels of breakthrough technologies such as CCS and use of alternative binders predict maximum emissions reduction potentials of 62–86% in cement production (DECC, 2015b; European Commission, 2018; MPA, 2015a). Roadmaps with net‐zero emissions pathways suggest these results are limited by focusing solely on cement production, that is, without considering decarbonization measures downstream in the CMs cycle (Favier et al., 2018; Material Economics, 2018). Net‐zero CMs cycle emissions are thought to be possible by combining breakthrough decarbonization measures, for example, kiln electrification, with multiple material efficiency measures along the CMs cycle (Figure 1). Integrated modeling of these various measures, including technological advancements which may potentially transform the CMs cycle, is needed to support effective industrial decarbonization policy (Habert et al., 2020).

## DISCUSSION

The European cement industry has made significant progress in reducing its emissions intensity through improved energy efficiency and use of alternative solid fuels between 2000 and 2020. However, achieving decarbonization requires significant technological developments and disruptive change to the current CMs cycle, including both technical as well as non‐technical changes, including behavioral. Currently, the focus of industry and policy in Europe in achieving decarbonization mainly relies on reducing emissions from cement production via CCS and alternative cements rather than reducing cement demand by systematically improving material efficiency. The latter approach requires more nuanced input and support from policy makers, which can be facilitated by more clearly communicating the various measures available and their decarbonization potentials.

Here, we reviewed the literature related to cement and decarbonization to combine various proposed energy, emissions, and material efficiency measures into a single visualization (Figure 1) that presents an overview of their range, estimated decarbonization potentials, and the diversity of actors involved in implementing them. The visualization may be improved by incorporating barriers associated with deployment of the reviewed decarbonization measures and existing policies targeting these barriers. We expect that this will facilitate the identification of gaps in cement and industrial decarbonization policy, and thus development of improved policy measures. For example, the introduction of embodied emissions of a structure kg CO_2_‐eq./m^2^ structure as a criterion on which to award public construction contracts may encourage design optimization and reduce overspecification (Favier et al., 2018). Development and uptake of performance‐based standards, enabling free choice of material as long as the required performance indicators for the component or product that it is embedded in is met, is another policy measure that may significantly affect various activities along the CMs cycle and reduce its overall emissions.

The literature contributing to the quantitative estimates in our annotated CMs cycle (Figure 1) can be improved by using more consistent terminology and units in defining decarbonization measures and their emissions reduction potentials. This is especially important in analysis of material efficiency measures, since they span the CMs cycle and so their decarbonization potentials may refer to various products (PC clinker, concrete, etc.). The “binder intensity” concept, that is, kg binder/m^3^ concrete and MPa concrete compressive strength (Damineli et al., 2010), is one attempt to standardize such comparative analyses, by relating the (volume specific) contribution of the binder to arguably the most important functional property of CMs, compressive strength. However, the applicability of “binder intensity” is specific to materials such as concrete that contain a binder component, which is thus limited in comparing different types of materials (concrete, steel, timber, etc.). Binder intensity and other similar indicators should thus be accompanied by their underlying mass and/or volume and/or density data. This would enable their conversion into mass units and used more generically, for example, to quantify stocks and flows in material cycles, life cycle assessment studies, etc. It is additionally important because “binder intensity” alone does not account for differences in binder type (e.g., PC binder, alkali‐activated binders), which may have substantially different embodied emissions.

Currently, definitions of material efficiency measures vary. Consensus in categorizing material efficiency measures is needed to improve clarity in their cumulative decarbonization potential and to facilitate quantitative comparisons among measures. For example, Shanks et al.’s definition of over‐specification includes matching concrete classes to concrete element requirements and excess concrete used in inefficient structural design (Shanks et al., 2019). On the other hand, Favier et al.’s definition treats these measures separately (Favier et al., 2018). We recommend the latter definition, which we feel is clearer due to the complexities of specification and design at both material and structure levels.

Finally, disaggregating CMs demand by application facilitates targeted policies focusing downstream of cement production, and thus towards material efficiency. An example policy outcome could be an increase in the proportion of concrete structures, of certain exposure classes, that contain specific cementitious binder types (chosen to match the specific exposure class), rather than a generic or “all‐purpose” cementitious binder (e.g., CEM I). Such a policy would require greater data availability of the cementitious binder types and concrete exposure classes used in different applications, so that they can be matched in an efficient manner. These data would facilitate identification of applications that have high cement, mortar, and/or concrete demand as well as relatively inefficient use of these materials. Extending this disaggregation of CMs by application temporally and to the use and end‐of‐life stages of the CMs cycle would enable further targeted policies, such as improving material efficiency in structural repairs. The various actors along the CMs cycle, including industry associations, have a key role to play in making these data available.

## CONCLUSION

We have presented a concise visualization of the CMs cycle, annotated with some of its key stocks and flows, emissions, decarbonization measures, and their actors, from raw materials extraction through to end‐of‐life. It includes a semi‐quantitative breakdown of the contributions of each main process to total CMs cycle emissions, and the masses of cement to its various end uses, where available in the literature. We further reviewed this literature to summarize current estimates of the potentials for decarbonization measures to reduce CMs cycle emissions, which we distinguished in terms of energy efficiency, emissions efficiency, and material efficiency measures. The measures with the highest CMs cycle decarbonization potentials were found to be alternative binders (<∼28%), kiln electrification (∼23%), CCS (∼21%), cementitious substitution (<∼21%), and reduce overspecification of products (<∼13%). However, we found various high performance materials and improved engineering design measures, including use of pre‐cast manufacturing and 3D printing, that have the potential to transform the current CMs cycle and so were not able to be quantified in terms of their decarbonization potentials. The decarbonization potentials of end‐of‐life processes were found to be poorly quantified, which may lead to reductions or increases in CMs cycle emissions; recycling and downcycling of end‐of‐life concrete may not be environmentally beneficial. Therefore, we clarify the significance of measures to decarbonize the CMs cycle and provide a systematic basis to highlight technical barriers, stakeholder responsibilities, and policy gaps to be addressed to achieve industrial decarbonization.

## Supplementary Information

Supporting Information S1: This supporting information S1 provides the emission and decarbonization calculations and the data used to create Figure 1 in the main text.

Supporting Information S2: This supporting information S2 provides descriptions of the calculations of cementitious materials cycle emissions and decarbonization potentials shown in Figure 1 of the main text.
