# Supplementary material for: Decarbonizing the cementitious materials cycle: A whole‐systems review of measures to decarbonize the cement supply chain in the UK and European contexts
Source: J Ind Ecol. 2021 Feb 3;25(2):359–76. doi: 10.1111/jiec.13105 (PMC13079544; doi:10.1111/jiec.13105)
Supplement: Supplementary file 2 — Supporting Information S2: This supporting information S2 provides descriptions of the calculations of cementitious materials cycle emissions and decarbonization potentials shown in Figure 1 of the main text. [file 44498_2021_2502009_MOESM2_ESM.pdf]

SUPPORTING INFORMATION FOR:

Pamenter, S. and R.J. Myers. 2020.Decarbonising the cementitious materials cycle: a whole-system review of measures to decarbonise the cement supply chain in the UK and European contexts. *Journal of Industrial Ecology.*

Summary

This supporting information S2 provides descriptions of the calculations of cementitious materials cycle emissions and decarbonisation potentials shown in Figure 1 of the main text.

Table of Contents

S1. Calculation of the cementitious materials cycle emissions shown in Figure 1 ....S2

S1.1. Positive emissions S3

S1.1.1. Extraction and production S3

S1.1.2. Manufacturing S4

S1.1.3. End-of-life S4

S1.1.4. Cementitious materials cycle........S5

S1.2. Negative emissions S6

S2. Calculation of the decarbonisation potentials shown in Figure 1........S6

S2.1. Decarbonisation potential calculation method........S7

S3. References in this supporting information file S9

S1. Calculation of the cementitious materials cycle emissions shown in Figure 1

We separated emissions into 'postive' emissions, that result in a net increase in global greenhouse gas (GHG) emissions, and 'negative' emissions, that result in a net decrease in global GHG emissions. We sum positive emissions along the entire cementitious materials cycle to 100% since there is currently relatively high uncertainty in the nature and extent of negative emissions from recarbonation. Therefore, values of negative emissions shown in Figure 1 are given in % of the total positive emissions throughout the entire cementitious materials cycle.

S1.1. Positive emissions

We combined results from multiple life cycle assessment studies to estimate relative emissions from each life cycle stage in the cementitious materials cycle. Due to the different goals and scopes of these studies (Teh et al., 2017; De Schepper et al., 2014), we adjusted their results to a common basis of cement with 75 mass% Portland cement clinker and 25 mass% non-clinkered cementitious materials, and concrete with 11 mass% Portland cement (representing the average European concrete). We assume that no positive emissions arise directly from the use stage in the cementitious materials cycle.

S1.1.1. Extraction and production

For the main processes of extraction and production in the cementitious materials cycle, we combined sub-process emissions reported in Teh et al. (2017) for 1 kg ordinary Portland cement. Extraction emissions arise from the following sub-processes: coal mining, road

transport, limestrone production, and 10% of the emissions from electricity generation. Production emissions arise from the following sub-processes: ordinary Portland cement clinker production (i.e., pyroprocessing emissions), other (e.g., petroleum and product manufacturing), and 90% of the emissions from electricity generation. The proportion of electricity generation emissions that we assigned to extraction (10%) and production (90%) is consistent with work by CEMBUREAU (2013). We thus estimated that 4% of emissions arise from extraction and 96% of emissions arise from production in the production of 1 kg ordinary Portland cement.

S1.1.2. Manufacturing

For the main process of manufacturing in the cementitious materials cycle, we combined emissions reported by Teh et al. (2017) for the production of 1 m^3 ordinary Portland cement concrete from the following sub-processes: concrete, electricity, road transport, and other emissions. We scaled emissions from the manufacturing process relative to emissions from extraction and production processes. Here, extraction includes gravel and sand sub-processes, as well as extraction emissions from cement production (defined in Section S1.2.1; 4% of combined extraction and production emissions). Production represents emissions from cement production (defined in Section S1.2.1; 94% of combined extraction and production emissions). We thus estimated that 6% of emissions arise from extraction, 75% of emissions arise from production, and 19% of emissions arise from manufacturing in the manufacturing of 1 m^3 ordinary Portland cement concrete.

S1.1.3. End-of-life

For the main process of end-of-life in the cementitious materials cycle, we used emissions calculated by De Schepper et al. (2014) from the entire life cycle of ordinary Portland cement concrete. We scaled emissions from extraction (6%), production (75%), and manufacturing (19%) processes (defined in Section S1.2.2) relative to the proportion of emissions for these processes as estimated in De Schepper et al. (2014), which was 90% of entire life cycle emissions (Figure 5). We thus estimate that 5% of emissions arise from extraction, 68% of emissions arise from production, 17% of emissions arise from manufacturing, and 10% of emissions arise from the end-of-life process in the life cycle of 1 m^3 ordinary Portland cement concrete.

S1.1.4. Cementitious materials cycle

We scaled positive emissions estimates for each main process (extraction, production, manufacturing, end-of-life), which are for 1 m^3 ordinary Portland cement concrete containing ~14 mass% cement, and which itself contains 95 mass% Portland cement clinker, to estimate the distribution of emissions for an average European concrete. This average European concrete contains 11 mass% cement, which itself contains ~75 mass% Portland cement clinker. Here, we assumed that NCMs are burden free, i.e., that no emissions arise from their production. We thus estimate that 6% of emissions arise from extraction, 57% of emissions arise from production, 23% of emissions arise from manufacturing, and 14% of emissions arise from the end-of-life process in cementitious materials cycle. Our estimate of emissions from the main process of production should be interpreted as a lower bound, since economic allocation of burdens associated with using non-clinkered cementitious materials (e.g. coal fly

ash, granulated blast furnace slag) in blended Portland cement concrete leads to minor contributions (~<10%) for these materials to their (cradle-to-gate) life cycle emissions (Teh et al., 2017).

S1.2. Negative emissions

We estimated the ranges (in %) of positive cementitious materials cycle emissions that are reabsorbed by concrete during use and at end-of-life.

For recarbonation during use, we took an average recarbonation extent in Denmark, Sweden, Norway, and Iceland, and assumed this value as representative of Europe (Pade & Guimaraes, 2007). Recarbonation at end-of-life varies significantly depending on the type of treatment. Given the variation in end-of-life treatments available, we calculated an upper and lower bound for end-of-life concrete recarbonation. The upper bound was based on crushing and recycling (90 mass% end-of-life concrete recycling rate), whereas the lower bound was based on landfilling (0 mass% end-of-life concrete recycling rate). Recarbonation extents were converted into % positive cementitious materials cycle emissions values by multiplying them with the % positive cementitious materials cycle emissions arising from limestone calcination and by applying a recarbonation factor (100% recarbonation equates to 75% process emissions reabsorbed).

We thus estimate that 7% of positive cementitious materials cycle emissions are reabsorbed during use, and 1-10% of positive cementitious material cycle emissions are reabsorbed at end-of-life.

S2. Calculation of the decarbonisation potentials shown in Figure 1

We gathered reported estimates of the potential emissions reductions that may result from applications of decarbonisation measures in the cementitious materials cycle. The scopes and methods used to develop these estimates differ, e.g., some cite emissions reductions as percentages of cement production while others give estimations across different parts of the cementitious materials cycle. Therefore, to compare the relative potentials of these decarbonisation measures, all estimates were converted into units of % positive cementitious materials cycle emissions. The estimates and their sources are shown in the ‘decarbonisation calculations’ worksheet in the Supporting Information spreadsheet. This worksheet also details the different assumptions and boundaries used in our calculations. We summarise our calculation method below (Section S2.1).

S2.1. Decarbonisation potential calculation method

We first identified the stages of the cementitious materials cycle that would be affected by a cited decarbonisation measure, e.g., extraction and production, production only, etc. We then calculated the % of positive cementitious materials cycle emissions that the identified stages accounted for, which is the % of emissions that may be affected by the decarbonisation measure. The cited decarbonisation potential was then used to calculate the % positive cementitious materials cycle emissions of the same stages, after application of the decarbonisation measure. These two values (before and after application of a decarbonisation measure) were then used to calculate the overall change in cementitious material cycle emissions from the application of a decarbonisation measure. Negative emissions remained unchanged in our calculations; however, in the Supporting Information spreadsheet

(decarbonisation calculations worksheet) we highlight decarbonisation measures that would reduce negative recarbonation emissions as well as positive emissions in the cementitious materials cycle.

S3. References in this supporting information file

CEMBUREAU (2013). The role of cement in the 2050 low carbon economy. Retrieved 13 December 2019, from https://cembureau.eu/media/1500/cembureau_2050roadmap_lowcarboneconomy_2013-09-01.pdf

De Schepper, M., Van den Heede, P., Van Driessche, I., & De Belie, N. (2014). Life cycle assessment of completely recyclable concrete. *Materials*, *7*(8), 6010-6027. https://doi.org/10.3390/ma7086010

ERMCO, 2019, Ready-mixed concrete industry statistics, year 2018. Retrieved 4 October 2020 from http://ermco.eu/new/wp-content/uploads/2020/08/ERMCO-Statistics-30.08.2019-R4-1.pdf

GCCA (2017). GNR project. Retrieved 13 February 2020, from https://gccassociation.org/gnr/

Pade, C., & Guimaraes, M. (2007). The CO_2 uptake of concrete in a 100 year perspective. *Cement and Concrete Research*, *37*(9), 1348-1356. https://doi.org/10.1016/j.cemconres.2007.06.009

Teh, S. H., Wiedmann, T., Castel, A., & de Burgh, J. (2017). Hybrid life cycle assessment of greenhouse gas emissions from cement, concrete and geopolymer concrete in Australia. *Journal of Cleaner Production*, *152*, 312-320. https://doi.org/10.1016/j.jclepro.2017.03.122
